# Uncovering non-linear dietary predictors of cardiovascular disease risk in older adults with periodontitis: a cross-sectional analysis

**DOI:** 10.3389/fnut.2026.1791821

**Published:** 2026-03-18

**Authors:** Jiefan Liu, Jinhao Wu, Xin Nie, Sunqiang Hu, Qiang Xu

**Affiliations:** 1 Department of Oral Maxillofacial Surgery, School and Hospital of Stomatology, Wenzhou Medical University, Wenzhou, Zhejiang, China; 2 Nursing Department, School and Hospital of Stomatology, Wenzhou Medical University, Wenzhou, Zhejiang, China

**Keywords:** aging, cardiovascular disease, dietary patterns, machine learning, periodontitis

## Abstract

**Objective:**

This study investigates whether the chronic inflammatory state associated with periodontitis alters the relationship between habitual diet and cardiovascular disease (CVD) risk in older adults.

**Methods:**

Data from three NHANES cycles (2009–2014) and the MyPyramid Equivalents Database (MPED) were integrated. To validate the generalizability of the findings, 183 older adults with periodontitis were included in an external validation study recruited from an independent hospital-based cohort. Feature selection was performed using correlation coefficients and the BORUTA algorithm. Six machine learning models were constructed, and SHAP and LIME algorithms were applied to interpret the associations between dietary trace elements, habitual food intake, and CVD risk.

**Results:**

The XGBoost model demonstrated superior predictive performance (Validation AUC-ROC: 0.854 for NHANES, 0.889 for MPED). SHAP analysis identified key protective factors against CVD in older adults with periodontitis. In the exploratory NHANES model, the top predictors included theobromine, lycopene, total sugar, food folate, beta-cryptoxanthin, and magnesium. The MPED model identified meat, whole grains, cured meat, tomatoes, eggs, added sugars, and total fruits as strong protective factors. Crucially, multivariable logistic regression analysis of the external cohort confirmed that higher consumption of red meat (OR = 0.46, *p* = 0.013) and sweets (OR = 0.11, *p* < 0.001) remained significantly associated with reduced CVD risk, whereas traditional antioxidant sources like green vegetables did not show statistical significance in this specific inflammatory population.

**Conclusion:**

Periodontitis-induced inflammation may partially invert dietary CVD risks, rendering higher intake of meat and sugars protective in this specific context. Interpretable XGBoost models reveal these non-linear effects, enabling more precise clinical nutrition guidance for older adults with periodontitis.

## Background

Periodontitis represents a critical public health burden in the aging population, characterized by chronic inflammation and the progressive destruction of tooth-supporting tissues. In the United States, approximately 62.3% of adults aged ≥65 years are affected, with nearly half exhibiting probing depths (PD) ≥ 4 mm and over 60% showing clinical attachment loss (AL) ≥ 5 mm (1). Globally, the prevalence remains alarmingly high; a systematic review in China reported pooled rates of 57.0% for PD ≥ 4 mm and 70.1% for AL ≥ 4 mm among older adults (1987–2015), with a noted male predilection (2). Aging exacerbates susceptibility to this condition through diminished healing capacity and immunosenescence (3). Crucially, periodontitis is not merely a localized oral pathology; it acts as a potent, modifiable amplifier of systemic cardiovascular disease (CVD) risk (4, 5). Recent evidence confirms that severe periodontitis significantly elevates the risk of cerebro-cardiovascular events, with hazard ratios reaching 1.16 in large-scale cohorts (4) and odds ratios (ORs) as high as 3.59 in cross-sectional analyses (5).

While dietary interventions are pivotal in CVD prevention, current guidelines are predominantly derived from general population cohorts. Substantial evidence demonstrates that diets rich in whole grains, fruits, legumes, and nuts reduce CVD mortality risk by 13–28%, whereas red and processed meats, as well as added sugars, are consistently associated with an increased risk of up to 23% (6, 7). Such protective effects are widely attributed to the synergistic action of dietary fiber, antioxidants, and key micronutrients—such as magnesium, selenium, and potassium—within the whole-food matrix (8–12). Meta-analyses confirm that a 100 mg/d increment in magnesium intake decreases stroke risk by 7% and heart failure by 22% (9). Conversely, deficiencies in selenium and zinc impair antioxidant defenses, thereby increasing CVD susceptibility (8).

However, a critical knowledge gap remains: conventional dietary recommendations may be ill-suited for older adults burdened by periodontitis-induced systemic inflammation. The complex interplay between whole-food nutrients, dosage, and CVD risk remains largely unexplored in this high-inflammation demographic. We hypothesize that the chronic systemic inflammation and oxidative stress accompanying periodontitis—driven by NLRP3 inflammasome activation and reactive oxygen species (ROS) accumulation (13, 14)—may fundamentally alter nutrient metabolism and bioavailability. Consequently, this inflammatory milieu could modify, or even invert, the established associations between diet and CVD outcomes. For a cohort where periodontitis prevalence approaches 70% (15), a re-evaluation of the dietary framework is imperative.

Standard linear statistical models, which underpin current dietary guidelines, are ill-equipped to capture the complex, non-linear interactions between nutrients and disease in such a pro-inflammatory state. To address this, the present study integrated Machine Learning (ML) models—specifically designed to resolve high-dimensional, non-linear data structures—using data from the National Health and Nutrition Examination Survey (NHANES) and the MyPyramid Equivalents Database (MPED). Our objectives were twofold: (i) to construct a robust predictive model for CVD risk in older adults (>50 years) with periodontitis; and (ii) to employ interpretable algorithms (SHAP, LIME) to identify key modifiable dietary factors and elucidate their non-linear contributions. Through this approach, we aim to uncover novel, precision-nutrition strategies to mitigate CVD risk in this vulnerable population.

## Materials and methods

### Study population

**Figure 1:**
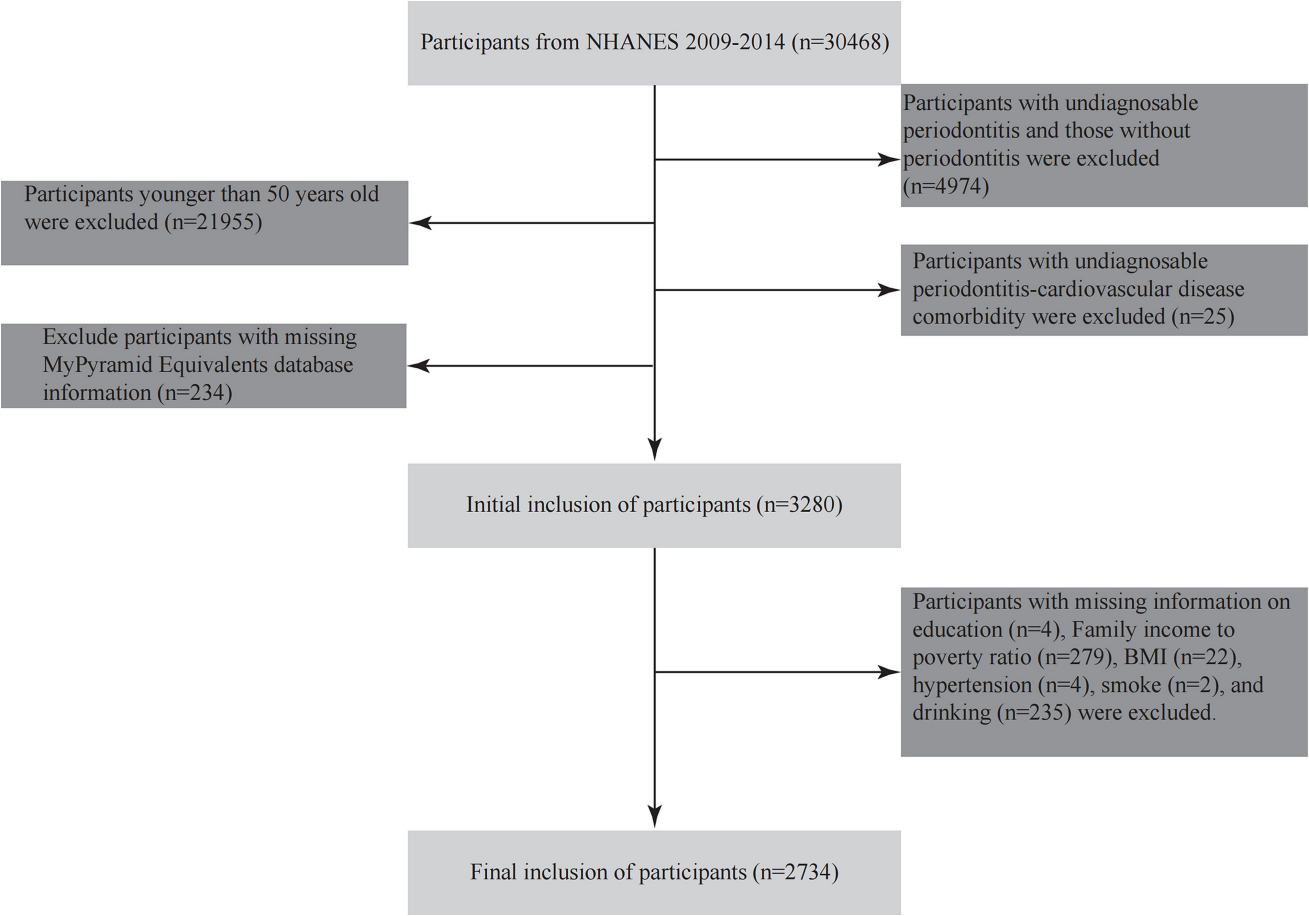
Flowchart of participant inclusion.

This study utilized data from three continuous cycles of the National Health and Nutrition Examination Survey (NHANES, 2009–2014), a nationally representative cross-sectional survey conducted by the National Center for Health Statistics (NCHS). From an initial pool of 30,468 participants, we established a final analytical sample of 2,734 older adults with confirmed periodontitis (Figure 1). Exclusion criteria were as follows: (1) age <50 years (*n* = 21,955); (2) no periodontal examination data or absence of periodontitis (*n* = 4,974); (3) missing data on CVD history (*n* = 25); (4) incomplete dietary recall data (*n* = 234); and (5) missing values for key covariates, including education, poverty income ratio (PIR), or smoking status (*n* = 546).

### Assessment of dietary exposures

Dietary intake was assessed using two 24-h dietary recall interviews (the first conducted in-person and the second by telephone) utilizing the USDA Automated Multiple-Pass Method. Micronutrient data were extracted from the NHANES Dietary Interview files. To estimate daily food group consumption, NHANES food codes were linked to the USDA MyPyramid Equivalents Database (MPED).

### Diagnosis of periodontitis and cardiovascular disease

Periodontitis was diagnosed according to the CDC/AAP case definitions based on a full-mouth periodontal examination, which measured clinical attachment loss (AL) and probing depth (PD) at six sites per tooth for up to 28 teeth (15). CVD status was defined based on self-reported physician diagnoses of heart failure, coronary heart disease, angina, myocardial infarction, or stroke, as recorded in the Medical Conditions Questionnaire.

### Covariates

We incorporated a comprehensive set of sociodemographic and clinical covariates, including age, sex, race/ethnicity, education level, family income-to-poverty ratio (PIR), body mass index (BMI), smoking status (never, former, or current), and alcohol consumption. Hypertension and diabetes were defined using a composite of self-reported diagnosis, medication use, and objective laboratory thresholds (HbA1c, fasting plasma glucose, or oral glucose tolerance test).

### External clinical validation cohort

To verify the generalizability of the ML-derived findings, we conducted a cross-sectional validation study at at our department, from November 2025 to January 2026. This study was approved by the Ethics Committee of Wenzhou Medical University (Ethics Review No. 2026-011, School of Stomatology, Wenzhou Medical University). The inclusion criteria mirrored those of the NHANES dataset.

Dietary intake was assessed using a simplified semi-quantitative Food Frequency Questionnaire (SQ-FFQ). To ensure methodological validity while maintaining clinical feasibility, frequency categories were adapted from validated questionnaires used in the China Kadoorie Biobank (CKB) study (16). Participants reported their consumption frequency of key food items identified by our SHAP analysis using a standard 5-point scale (1 = Never/Rarely to 5 = Daily).

### Statistical analysis

Baseline characteristics were compared using Student’s t-tests for continuous variables and Chi-square tests for categorical variables. For the external validation cohort, multivariable logistic regression models were constructed to calculate adjusted ORs and 95% confidence intervals for CVD risk, controlling for age, sex, smoking status, and periodontal severity. All analyses were performed using IBM SPSS 24.0 and R 4.3.0, with statistical significance defined as *p* < 0.05.

### Machine learning model development and interpretation

A total of 90 features (46 NHANES micronutrients, 37 MPED components, 7 categorical variables) were considered. A multicolinerity issue was corrected by dropping features with Spearman |*ρ*| > 0.9. SMOTE was used to balance CVD outcome class imbalance. All variables were normalized. The importance of features was determined using the Boruta algorithm (500 iterations, random forest base); only” confirmed” features were used. The dataset was split 7:3 into training and validation sets. Six ML models were developed using MLR3: Random Forest, LightGBM, K-Nearest Neighbors, Naive Bayes, SVM, and XGBoost. Performance was assessed via accuracy, F-beta, AUC-ROC (primary), sensitivity, specificity, and AUC-PR, with 10-fold cross-validation. Differences among models were evaluated with ANOVA, Kruskal–Wallis. In order to probe whether the presence of periodontitis-induced inflammation linearly modifies the relationship between risk-nutrients and CVD outcomes, we then aimed to rank model interpretability using SHapley Additive exPlanations (SHAP) to see both global and interaction effects, and Local Interpretable Model-agnostic Explanations (LIME) to see local prediction transparency.

## Results

### Baseline characteristics

The final analytical sample comprised 2,734 older adults with periodontitis (mean age 63.89 ± 9.17 years), of whom 59.1% were female. CVD was identified in 334 participants (12.2%), who were significantly older than those without CVD (mean age 68.42 ± 9.26 years). Compared to the non-CVD group, participants with CVD consumed significantly less energy (*p* = 0.003), protein (*p* = 0.034), carbohydrates (*p* = 0.002), and total sugars (*p* = 0.002). Specific food group analysis revealed significantly lower intake of dark green vegetables (*p* = 0.003), meat (*p* = 0.039), added sugars (*p* = 0.002), and alcoholic beverages (*p* = 0.042) in the CVD group (Table 1).

**Table 1:** Baseline demographic, clinical, and dietary characteristics of the study population (NHANES 2009–2014), stratified by cardiovascular disease (CVD) status.

Characteristic	*N*	Overall (*n* = 2,734)	Non-CVD (*n* = 2,400)	CVD (*n* = 334)	*P*
Sex^b^, *n* (%)	2,734				**<0.001**
Female		1,617 (59.14%)	1,388 (57.83%)	229 (68.56%)	
Male		1,117 (40.86%)	1,012 (42.17%)	105 (31.44%)	
Age^a^, Mean ± SD	2,734	63.89 ± 9.17	63.26 ± 8.97	68.42 ± 9.26	**<0.001**
Race/ethnicity^b^, *n* (%)	2,734				**<0.001**
Mexican		407 (14.89%)	377 (15.71%)	30 (8.98%)	
Other Hispanic		259 (9.47%)	236 (9.83%)	23 (6.89%)	
Non-Hispanic White		1,167 (42.68%)	980 (40.83%)	187 (55.99%)	
Non-Hispanic Black		685 (25.05%)	607 (25.29%)	78 (23.35%)	
Other race		216 (7.90%)	200 (8.33%)	16 (4.79%)	
Education^b^, *n* (%)	2,734				0.603
<9th grade		372 (13.61%)	322 (13.42%)	50 (14.97%)	
9–11th grade		427 (15.62%)	369 (15.38%)	58 (17.37%)	
High school diploma/GED		644 (23.56%)	566 (23.58%)	78 (23.35%)	
Some college/AA degree		749 (27.40%)	658 (27.42%)	91 (27.25%)	
≥College graduate		542 (19.82%)	485 (20.21%)	57 (17.07%)	
Family income to poverty ratio^a^, Mean ± SD	2,734	2.48 ± 1.58	2.52 ± 1.58	2.25 ± 1.52	**0.003**
BMI^a^, Mean ± SD	2,734	29.36 ± 6.32	29.16 ± 6.20	30.79 ± 6.99	**<0.001**
Smoking status^b^, *n* (%)	2,734				0.683
No		2,192 (80.18%)	1,927 (80.29%)	265 (79.34%)	
Yes		542 (19.82%)	473 (19.71%)	69 (20.66%)	
Hypertension^b^, *n* (%)	2,734				**<0.001**
No		1,277 (46.71%)	1,197 (49.88%)	80 (23.95%)	
Yes		1,457 (53.29%)	1,203 (50.13%)	254 (76.05%)	
Diabetes^b^, *n* (%)	2,734				**<0.001**
No		1,859 (68.00%)	1,674 (69.75%)	185 (55.39%)	
Yes		609 (22.28%)	498 (20.75%)	111 (33.23%)	
Energy^a^, Mean ± SD	2,734	2,004.06 ± 908.50	2,020.34 ± 907.57	1,887.09 ± 907.93	**0.003**
Protein^a^, Mean ± SD	2,734	79.73 ± 41.06	80.23 ± 41.00	76.12 ± 41.39	**0.034**
Carbohydrate^a^, Mean ± SD	2,734	239.81 ± 109.86	241.94 ± 109.78	224.46 ± 109.37	**0.002**
Total sugar^a^, Mean ± SD	2,734	102.59 ± 65.93	103.67 ± 65.57	94.82 ± 68.02	**0.002**
Dietary fiber^a^, Mean ± SD	2,734	17.40 ± 10.90	17.61 ± 11.08	15.93 ± 9.32	**0.022**
Added sugars^a^, Mean ± SD	2,734	14.76 ± 13.60	14.93 ± 13.47	13.52 ± 14.46	**0.002**
Meat^a^, Mean ± SD	2,734	1.60 ± 2.58	1.59 ± 2.62	1.66 ± 2.32	**0.039**
Dark green vegetables^a^, Mean ± SD	2,734	0.14 ± 0.41	0.15 ± 0.42	0.09 ± 0.30	**0.003**
Food folate^a^, Mean ± SD	2,734	224.71 ± 154.37	228.77 ± 158.55	195.55 ± 116.28	**<0.001**
Magnesium^a^, Mean ± SD	2,734	296.69 ± 145.70	300.41 ± 148.40	269.93 ± 121.50	**0.001**
Alcoholic beverages^a^, Mean ± SD	2,734	0.73 ± 2.07	0.76 ± 2.10	0.55 ± 1.86	**0.042**

a Student *t*-test.

b Chi-square test.

SD, standard deviation; Full breakdown of dietary micronutrients and additional food groups is provided in Supplementary Table S1. Bold values indicate *P* < 0.05.

### Feature selection for machine learning

**Figure 2:**
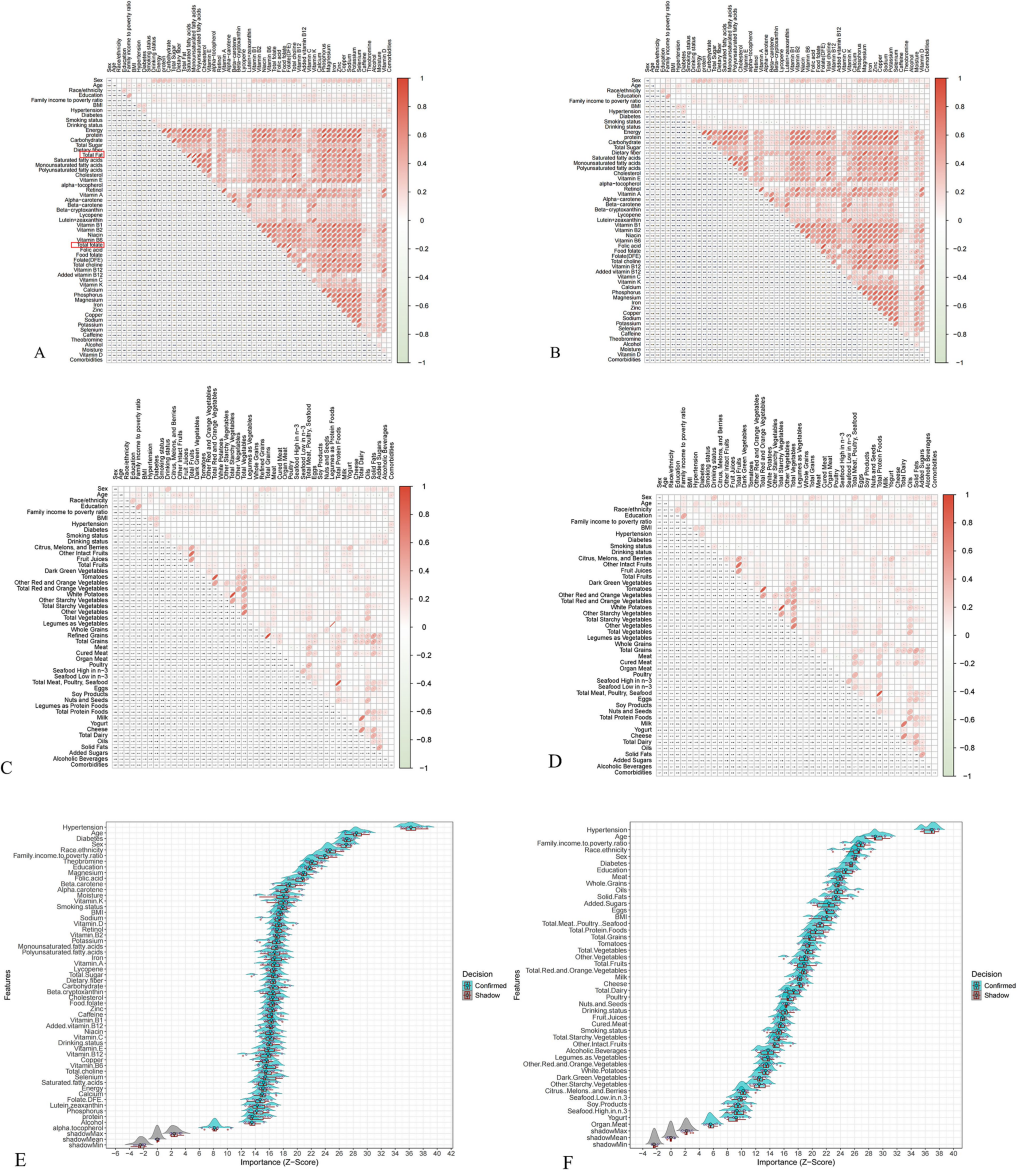
Feature selection and multicollinearity management. **(A)** NHANES dataset before removing collinearity; **(B)** NHANES dataset after removing collinearity; **(C)** MPED before removing collinearity; **(D)** MPED after removing collinearity; **(E)** Feature importance for the NHANES dataset; **(F)** Feature importance for the MPED dataset.

To mitigate multicollinearity, features with Spearman correlation coefficients >0.9 were excluded, resulting in the removal of “Total Fat” and “Total Folate” from the NHANES dataset (Figures 2A,B), and “Legumes as Protein Foods” and “Refined Grains” from the MPED dataset (Figures 2C,D). Subsequent feature selection using the Boruta algorithm identified 54 NHANES micronutrients and 45 MPED food components as significantly associated with CVD risk (Figures 2E,F). These confirmed features were retained for model construction. The stability of Z-scores during the selection process is illustrated in Supplementary Figure S1.

### Model construction and evaluation

**Figure 3:**
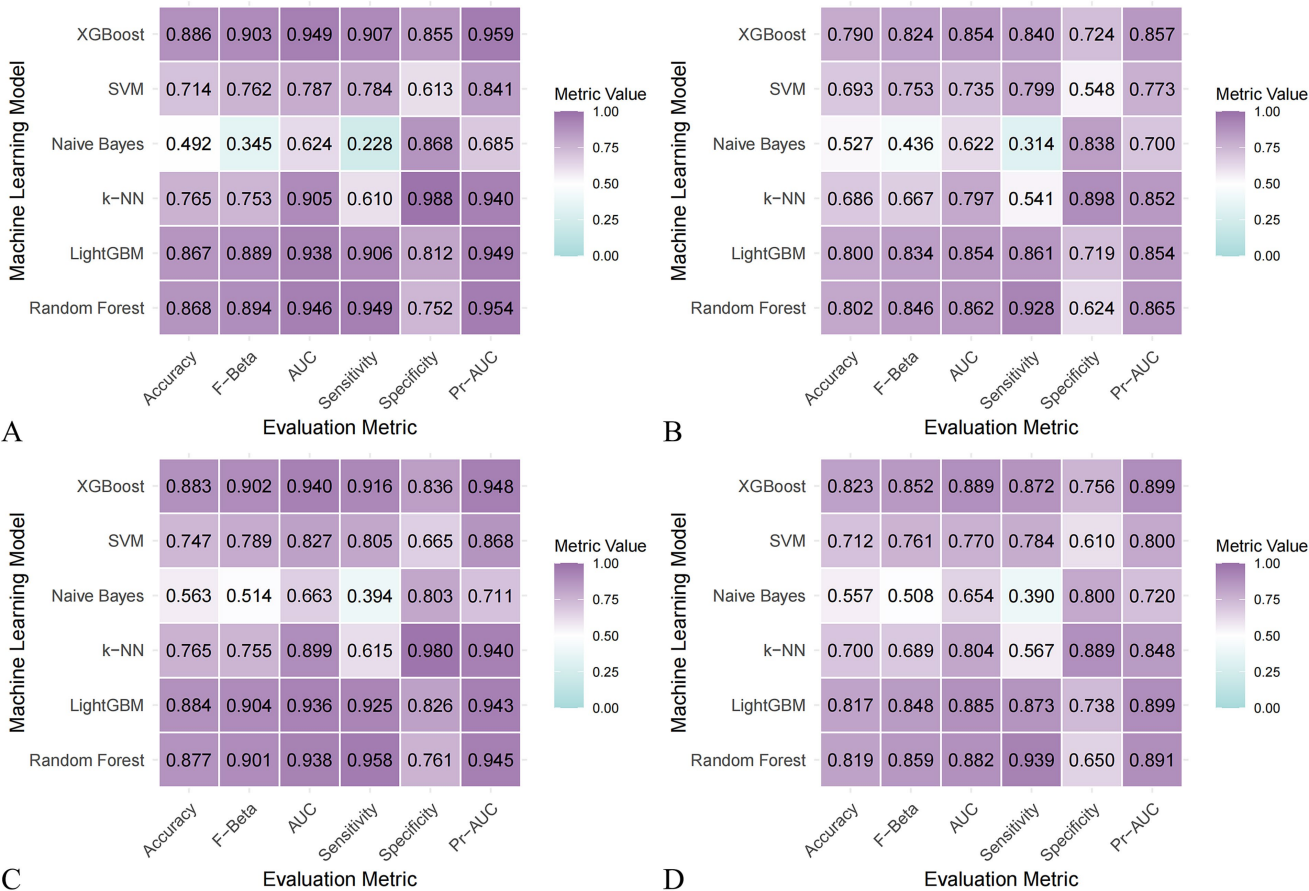
Heatmaps comparing the performance of different machine learning models in NHANES and the MPED. **(A)** NHANES training set; **(B)** NHANES validation set; **(C)** MPED training set; **(D)** MPED validation set.

**Figure 4:**
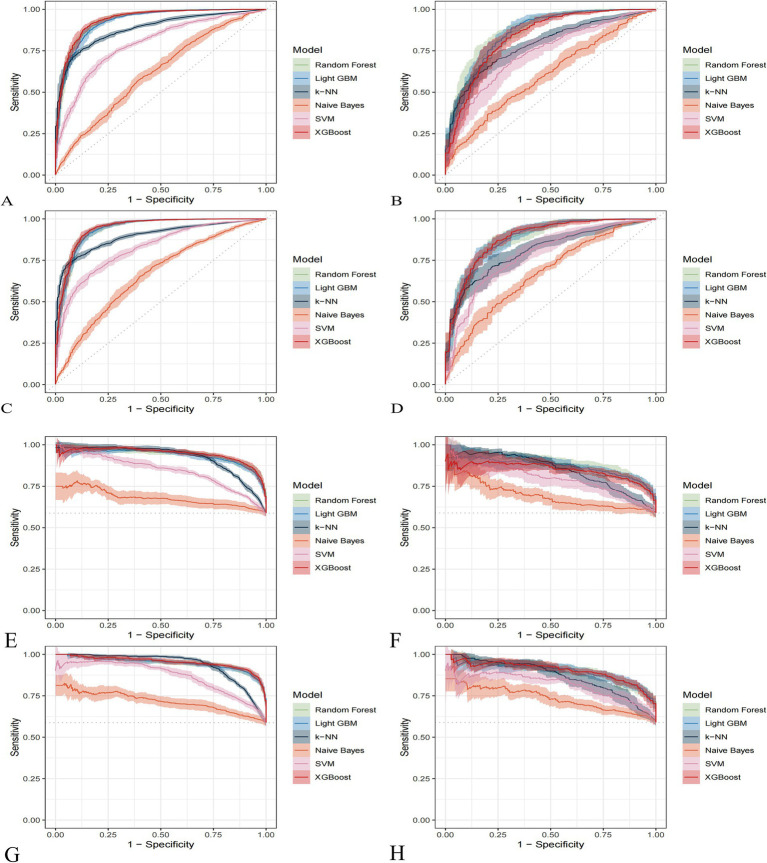
Comprehensive performance evaluation of six machine learning models. **(A–D)** Receiver operating characteristic curves illustrating the diagnostic ability of the classifiers. **(A)** NHANES training set; **(B)** NHANES validation set; **(C)** MPED training set; **(D)** MPED validation set. **(E–H)** Precision-recall curves evaluating model performance. **(E)** NHANES training set; **(F)** NHANES validation set; **(G)** MPED training set; **(H)** MPED validation set.

Comparative analysis of the six machine learning algorithms—visualized via performance heatmaps (Figure 3) and ROC curves (Figures 4A–D)—demonstrated that XGBoost achieved superior predictive performance across both datasets. For the NHANES dataset, the XGBoost model yielded a validation AUC-ROC of 0.854, with an accuracy of 0.790 and F-beta of 0.824 (Table 2). Training set metrics indicated robust model fitting without significant overfitting (Supplementary Table S2). Similarly, the model demonstrated high stability in the MPED dataset, achieving a validation AUC-ROC of 0.889, an accuracy of 0.823, and F-beta of 0.852 (Table 3; training metrics in Supplementary Table S3). Further benchmark analysis using Precision-Recall curves (Figures 4E–H) and statistical comparisons confirmed that the performance advantages of XGBoost over other models were statistically significant (*p* < 0.001; Supplementary Figures S2, S3).

**Table 2:** Benchmark comparison results for the NHANES validation set.

Model	Accuracy	F beta	Area under the ROC curve	Sensitivity	Specificity	Area under the PR curve
Random Forest	0.802 (0.772–0.833)	0.846 (0.820–0.872)	0.862 (0.839–0.885)	0.928 (0.905–0.951)	0.624 (0.567–0.682)	0.865 (0.826–0.904)
Light GBM	0.800 (0.781–0.818)	0.834 (0.817–0.851)	0.854 (0.836–0.872)	0.861 (0.822–0.899)	0.719 (0.677–0.760)	0.854 (0.816–0.893)
K-KNN	0.686 (0.661–0.712)	0.667 (0.630–0.705)	0.797 (0.772–0.823)	0.541 (0.489–0.593)	0.898 (0.867–0.930)	0.852 (0.823–0.882)
Naive Bayes	0.527 (0.492–0.562)	0.436 (0.387–0.485)	0.622 (0.581–0.664)	0.314 (0.267–0.362)	0.838 (0.799–0.876)	0.700 (0.653–0.746)
SVM	0.693 (0.660–0.725)	0.753 (0.725–0.781)	0.735 (0.708–0.762)	0.799 (0.761–0.837)	0.548 (0.473–0.622)	0.773 (0.724–0.822)
XGBoost	0.790 (0.771–0.809)	0.824 (0.804–0.844)	0.854 (0.832–0.875)	0.840 (0.812–0.868)	0.724 (0.678–0.771)	0.857 (0.820–0.894)
*P*	<0.001^a^	<0.001^a^	<0.001^b^	<0.001^a^	<0.001^a^	<0.001^a^

a ANOVA test.

b Kruskal-Wallis.

**Table 3:** Benchmark comparison results for the MyPyramid Equivalents Database validation set.

Model	Accuracy	F beta	Area under the ROC curve	Sensitivity	Specificity	Area under the PR curve
Random Forest	0.819 (0.789–0.850)	0.859 (0.833–0.884)	0.882 (0.856–0.908)	0.939 (0.915–0.963)	0.650 (0.603–0.697)	0.891 (0.862–0.919)
Light GBM	0.817 (0.783–0.851)	0.848 (0.820–0.877)	0.885 (0.855–0.914)	0.873 (0.851–0.895)	0.738 (0.678–0.797)	0.899 (0.868–0.929)
K-KNN	0.700 (0.665–0.736)	0.689 (0.650–0.727)	0.804 (0.779–0.829)	0.567 (0.519–0.615)	0.889 (0.854–0.924)	0.848 (0.817–0.879)
Naive Bayes	0.557 (0.527–0.588)	0.508 (0.479–0.537)	0.654 (0.615–0.694)	0.390 (0.356–0.425)	0.800 (0.752–0.848)	0.720 (0.672–0.767)
SVM	0.712 (0.674–0.749)	0.761 (0.725–0.796)	0.770 (0.743–0.797)	0.784 (0.751–0.817)	0.610 (0.551–0.669)	0.800 (0.770–0.829)
XGBoost	0.823 (0.796–0.851)	0.852 (0.828–0.877)	0.889 (0.863–0.915)	0.872 (0.853–0.891)	0.756 (0.705–0.806)	0.899 (0.869–0.929)
*P*	<.001^a^	<.001^a^	<.001^b^	<.001^a^	<.001^a^	<.001^a^

a ANOVA test.

b Kruskal-Wallis.

### SHAP and LIME interpretation of dietary predictors

**Figure 5:**
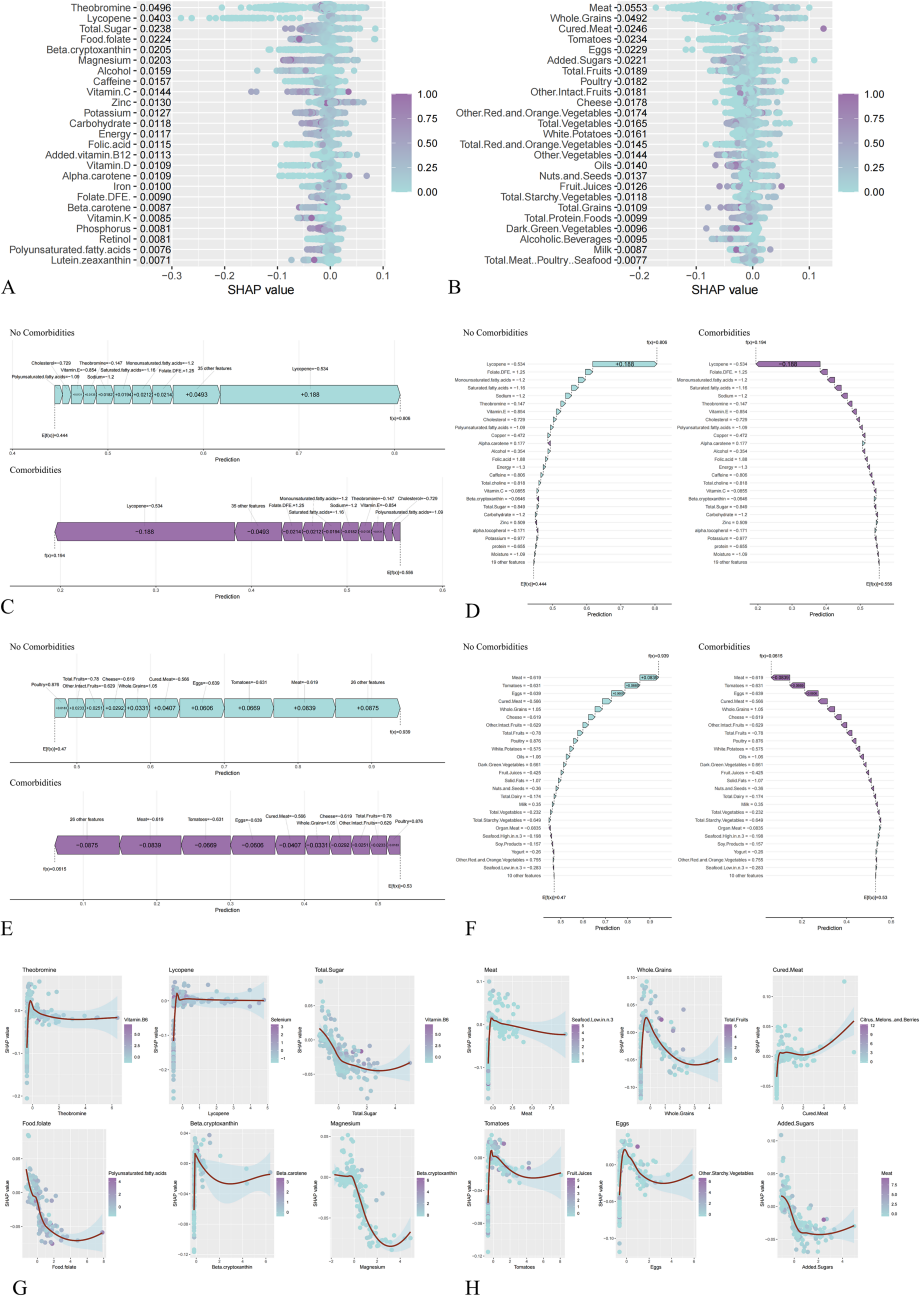
Multi-perspective interpretation of the XGBoost model using SHAP algorithms. **(A,B)** SHAP summary plots illustrating global feature importance. Features are ranked by descending importance on the *y*-axis. The *x*-axis represents the SHAP value (impact on model output). **(A)** NHANES; **(B)** MPED. **(C–F)** Individual-level explanations using force and waterfall plots for representative cases. **(C)** NHANES force plots; **(D)** NHANES waterfall plots; **(E)** MPED force plots; **(F)** MPED waterfall plots. **(G,H)** SHAP dependence plots depicting the non-linear relationships between the top six influential dietary features and CVD risk. **(G)** NHANES; **(H)** MPED.

To interpret the optimal XGBoost model, we applied SHAP and LIME algorithms. SHAP summary plots identified the top predictors of CVD risk. In the NHANES model, Theobromine, Lycopene, Total Sugar, Food Folate, Beta-cryptoxanthin, and Magnesium emerged as the strongest protective factors (Figure 5A). In the MPED model, Meat, Whole Grains, Cured Meat, Tomatoes, Eggs, Added Sugars, and Total Fruits were identified as key protective dietary components (Figure 5B). Individual-level predictions were visualized using Force plots, which illustrated how specific feature combinations shifted the baseline probability of being CVD-free (Figures 5C–F). SHAP dependence plots further elucidated the non-linear relationships between the top six influential features and CVD risk (Figures 5G,H).

**Figure 6:**
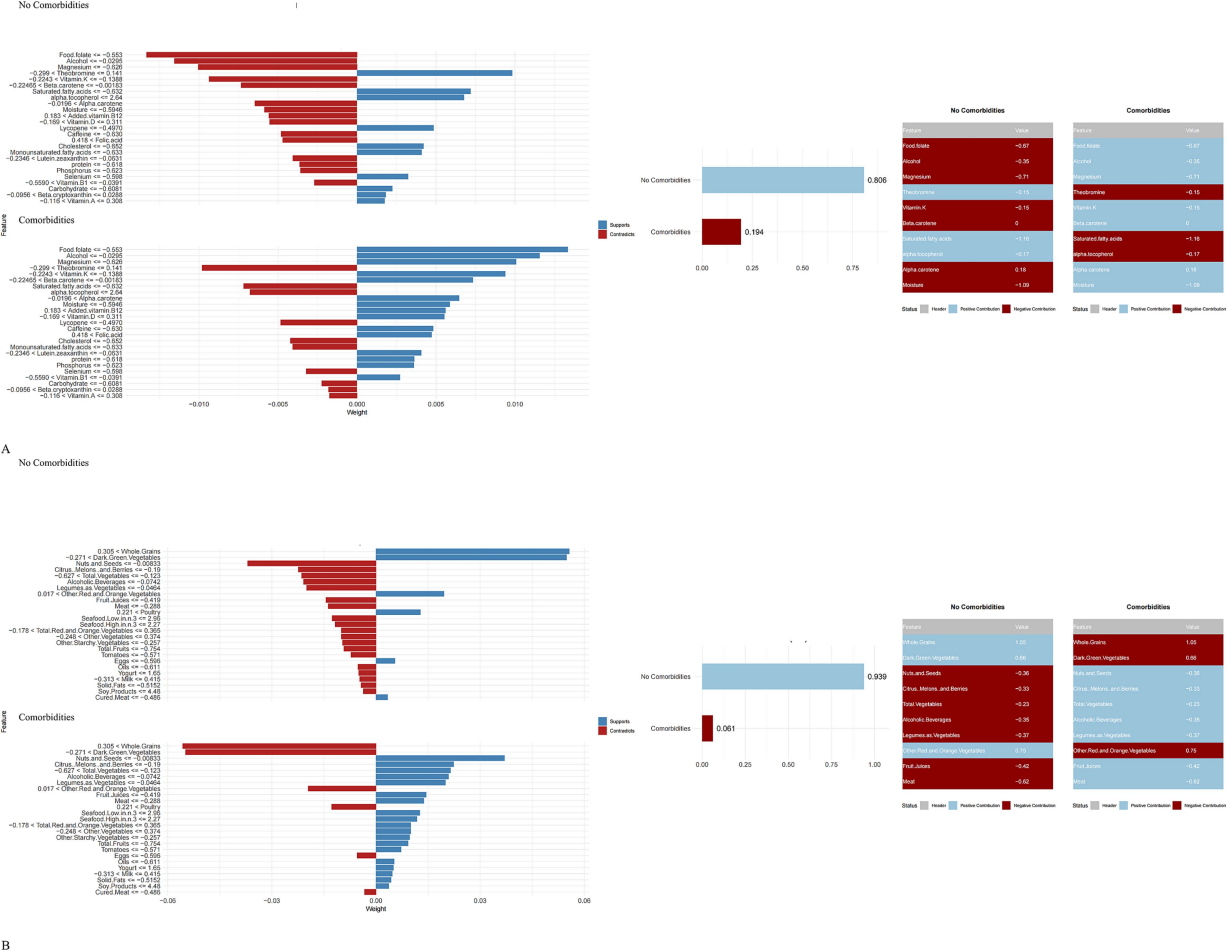
LIME-based interpretation of the best-performing machine learning model **(A,B)**. NHANES; **(B)** MyPyramid Equivalents Database.

Complementing this, LIME analysis provided local interpretability for individual predictions. For a representative CVD-free individual in the NHANES model (predicted probability 0.806), protective contributions were observed for specific normalized ranges of Theobromine, Saturated Fatty Acids, and Alpha-Tocopherol (Figure 6A). Similarly, in the MPED model (predicted probability 0.939), protective effects were driven by specific intake levels of Whole Grains, Dark Green Vegetables, and Eggs (Figure 6B).

### Validation in the independent clinical cohort

The baseline characteristics of the external validation cohort (*n* = 183) are detailed in Table 4. Consistent with the primary NHANES analysis, the CVD group exhibited a significantly higher prevalence of smoking (*p* = 0.028).

**Table 4:** Demographic and dietary characteristics of older adults with periodontitis in the validation cohort (*n* = 183).

Variables	Total (*n* = 183)	Non-CVD (*n* = 154)	CVD (*n* = 29)	*P*
Age^a^, Mean ± SD	60.16 ± 6.28	59.81 ± 6.26	62.03 ± 6.11	0.080
Red meat^a^, Mean ± SD	3.62 ± 0.63	3.67 ± 0.61	3.34 ± 0.72	**0.029**
Whole grains^a^, Mean ± SD	3.49 ± 0.57	3.54 ± 0.57	3.24 ± 0.51	**0.010**
Processed meat^a^, Mean ± SD	3.49 ± 0.69	3.63 ± 0.59	2.72 ± 0.70	**<0.001**
Tomatoes^a^, Mean ± SD	3.46 ± 0.64	3.49 ± 0.63	3.34 ± 0.72	0.277
Eggs^a^, Mean ± SD	3.61 ± 0.61	3.66 ± 0.56	3.34 ± 0.77	**0.046**
Sweets^a^, Mean ± SD	3.49 ± 0.63	3.62 ± 0.53	2.79 ± 0.68	**<0.001**
Fruits^a^, Mean ± SD	3.59 ± 0.76	3.64 ± 0.75	3.31 ± 0.76	**0.030**
Citrus^a^, Mean ± SD	3.48 ± 0.78	3.49 ± 0.81	3.41 ± 0.63	0.617
Chocolate and Tea^a^, Mean ± SD	3.15 ± 0.82	3.21 ± 0.81	2.86 ± 0.79	**0.036**
Green vegetables^a^, Mean ± SD	3.56 ± 0.64	3.56 ± 0.62	3.55 ± 0.78	0.959
Sex^b^, *n* (%)				**0.150**
1	79 (43.17)	70 (45.45)	9 (31.03)	
2	104 (56.83)	84 (54.55)	20 (68.97)	
Smoking^b^, *n* (%)				**0.028**
0	79 (43.17)	60 (38.96)	19 (65.52)	
1	66 (36.07)	59 (38.31)	7 (24.14)	
2	38 (20.77)	35 (22.73)	3 (10.34)	
Perio status^b^, *n* (%)				**0.192**
1	53 (28.96)	41 (26.62)	12 (41.38)	
2	67 (36.61)	60 (38.96)	7 (24.14)	
3	63 (34.43)	53 (34.42)	10 (34.48)	

a Student *t*-test.

b Chi-square test.

SD, standard deviation. Bold values indicate *P* < 0.05.

**Figure 7:**
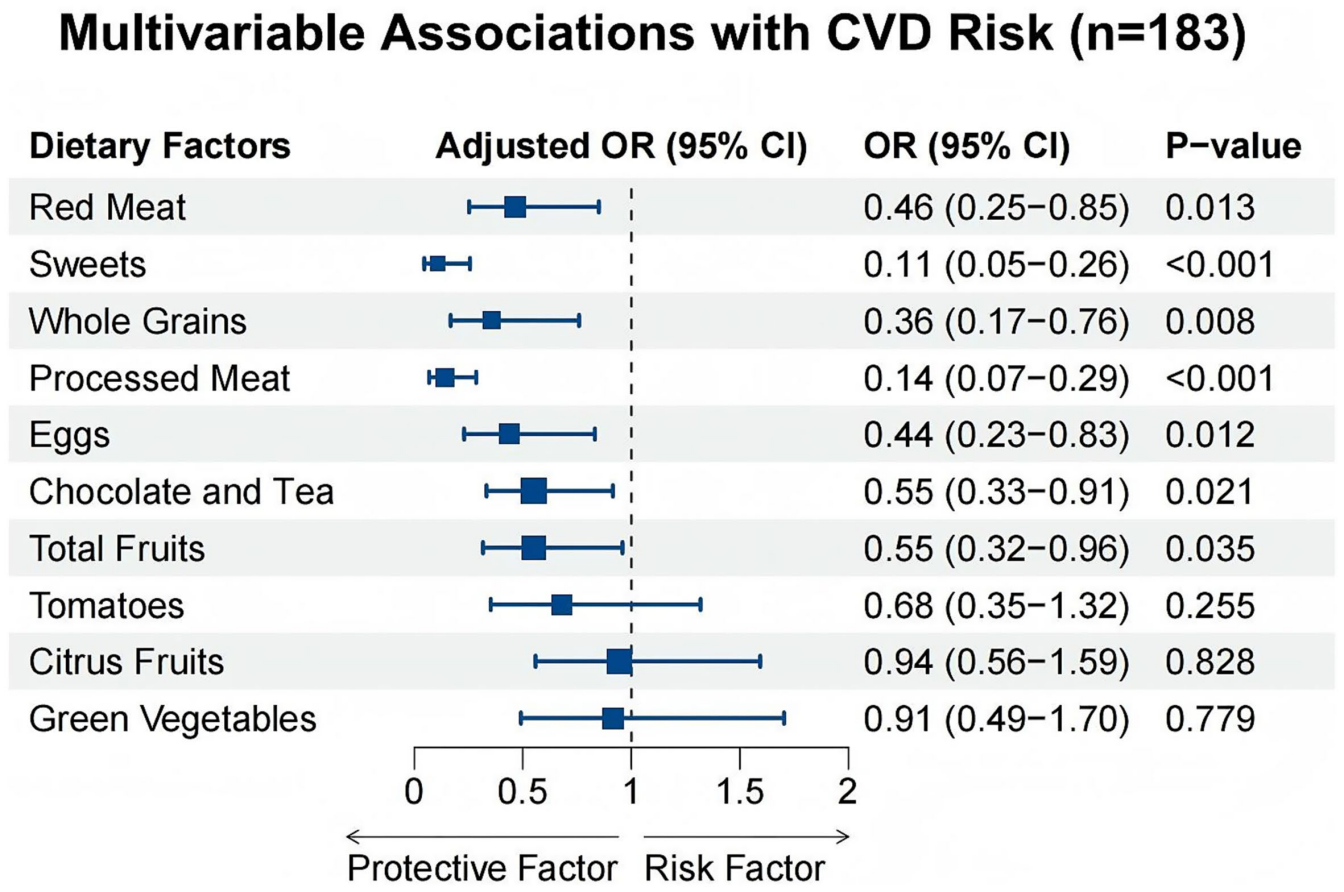
Forest plot summarizing the multivariable-adjusted associations between key dietary factors and CVD risk in the external clinical validation cohort (*n* = 183).

Multivariable logistic regression showed that higher consumption of Red Meat (Adjusted OR = 0.46, 95% CI: 0.25–0.85, *p* = 0.013) and Sweets (Adjusted OR = 0.11, 95% CI: 0.05–0.26, *p* < 0.001) was significantly associated with reduced CVD risk (Figure 7). Whole Grains, Processed Meat, Eggs, Chocolate and Tea, and Total Fruits were also verified as significant protective factors. In contrast, traditional antioxidant sources, including Green Vegetables, Tomatoes, and Citrus Fruits, showed no statistically significant association with CVD outcomes in this specific cohort.

## Discussion

Periodontitis represents a pervasive inflammatory burden in the aging population, affecting nearly two-thirds of individuals aged 65 and older (1, 15, 17). In this cohort, the disease acts not merely as a localized oral infection but as a critical, inflammation-driven amplifier of cardiovascular disease (CVD) risk (4, 5). This chronic inflammatory milieu may fundamentally alter the physiological response to dietary patterns, potentially rendering conventional nutritional paradigms—derived from the general population—less applicable (18–20). While NHANES data confirm the link between periodontitis and elevated CVD risk (OR 1.4–2.0) via biomarkers like CRP (5, 21, 22), the interaction between this inflammatory baseline and nutrient metabolism remains poorly understood. We hypothesized that the persistent inflammatory stress of periodontitis modulates metabolic systems, thereby necessitating a re-evaluation of dietary guidelines for this specific demographic.

Traditional linear statistical models are often ill-equipped to capture the high-dimensional, non-linear nutrient interactions inherent to this chronic inflammatory state. By leveraging an interpretable Machine Learning (ML) framework across the NHANES and MPED databases, our XGBoost models achieved high predictive accuracy (AUC-ROC 0.85–0.95) and, crucially, unveiled paradoxical nutrient–CVD relationships. Specifically, SHAP (which calculates the marginal contribution of each feature based on game theory) and LIME (a local surrogate model) provided transparent interpretations, where negative SHAP values explicitly indicated a protective directionality against CVD risk. Contrary to extensive epidemiological evidence linking red/processed meats (via TMAO production) and added sugars (via ROS accumulation) to increased CVD risk (23–26), our SHAP and LIME analyses identified these factors as significantly protective in older adults with periodontitis. This inversion of conventional dietary dogma suggests that the metabolic impact of these food groups is fundamentally reprogrammed by the underlying periodontal inflammation.

This discrepancy likely reflects unique physiological mechanisms driven by “inflammaging.” Periodontitis-induced NLRP3 inflammasome activation and oxidative stress create a distinct metabolic milieu (13, 14), where nutrient utilization may shift from homeostasis to survival. This discrepancy aligns with the established concept of “metabolic reprogramming.” Previous research indicates that the chronic IL-6–driven upregulation of hepcidin disrupts iron metabolism (27). In this inflammatory context, moderate intake of heme iron and zinc from meats may theoretically counteract inflammation-induced anemia and ferroptosis without promoting oxidative stress (28). Similarly, the apparent benefit of sugars might be twofold: first, providing rapid energy substrates to combat the catabolic state associated with chronic infection; and second, when derived from fiber-rich matrices (e.g., fruits), delivering polyphenols that suppress fructosamine formation and modulate the microbiota to reduce gingival NLRP3 activation (29, 30). While residual confounding from health behaviors cannot be ruled out, Mendelian randomization studies support the existence of such non-linear dose–response thresholds in inflammatory subgroups (5, 21).

Our model’s validity is further supported by its corroboration of known cardioprotective nutrients common to the general population. Magnesium emerged as a key protective factor, consistent with its role in endothelial stabilization and NLRP3 inhibition (11, 12, 20, 27). Likewise, the model correctly prioritized Food Folate for its capacity to promote homocysteine remethylation and improve endothelial function (31–33), and Lycopene for its potent ROS-scavenging properties (30, 34, 35). The identification of Beta-Cryptoxanthin and Theobromine aligns with literature demonstrating their lipid-modulating and mortality-reducing effects (36–40). Furthermore, the protective role of whole grains, mediated through fiber–polyphenol synergy (41–43), and the neutral-to-protective effects of eggs (likely due to choline content), indicate that our model captures the nuanced reality where nutrient-dense whole foods retain their essential value even amidst inflammation.

Interestingly, the integration of an external clinical validation cohort (*n* = 183) extended our findings from theoretical predictions to real-world verification. The clinical data provided supportive evidence of the ML results: red meats and sweets were significantly associated with protective effects, whereas green vegetables did not show statistical significance. This discrepancy reinforces the “metabolic reprogramming” hypothesis. Under the burden of systemic inflammation (e.g., elevated IL-6 and TNF-α), the aging body may prioritize readily available energy and high-quality proteins—such as simple carbohydrates and meat—to counteract the hypermetabolic state induced by chronic inflammation, thereby preserving muscle mass and endothelial resilience (44, 45). In this specific context, the subtle antioxidant effects of vegetables might be overshadowed by the pervasive inflammatory milieu. These findings validate our core argument that dietary guidelines for older adults with periodontitis should be disease-specific rather than a uniform application of general standards.

The primary clinical implication of this work is the necessity for precision nutrition guidelines tailored to periodontitis patients. Simply advising this vulnerable population to adopt a conventional “low-sugar, low-meat” diet may be suboptimal if it fails to account for the metabolic dysregulation caused by their inflammatory burden. Our interpretable ML approach represents a foundational step toward developing nuanced, disease-specific dietary strategies to optimize cardiovascular health in this high-risk group.

Nevertheless, several limitations should be noted. First, the cross-sectional nature of the NHANES data precludes causal inference and risks reverse causation, as the apparent metabolic protection of meat and sweets may merely reflect better masticatory ability rather than true dietary benefits. Second, the 24-h dietary recall method, used to generate both the NHANES micronutrient data and the MPED food group data, may introduce recall bias and underreport episodically consumed foods. Furthermore, methodological heterogeneity exists between the quantitative 24-h recall in NHANES and the frequency-based SQ-FFQ in our validation cohort, which precluded exact threshold calibration and restricted the validation to directional trends rather than absolute intake grams. Third, while the external validation cohort provided crucial clinical verification, its relatively small sample size (*n* = 183) and low number of CVD cases (*n* = 29) render the multivariable model underpowered, leading to wide confidence intervals and potential overfitting. Additionally, the external validation cohort lacked adjustment for socioeconomic status, which heavily influences dietary choices and may introduce residual confounding. Finally, although we adjusted for major confounders, the temporal relationship between periodontitis progression and CVD onset cannot be definitively established, and residual confounding from unmeasured factors (e.g., specific oral hygiene practices or statin use) may persist. Therefore, our findings warrant validation in larger, longitudinal, biomarker-based studies designed to prospectively test these unique dietary mechanisms.

## Conclusion

By integrating interpretable machine learning with clinical validation, we demonstrate that the chronic systemic inflammatory milieu associated with periodontitis may fundamentally invert established nutrient–CVD risks, rendering conventionally restricted foods—such as red meat and sweets—protective in this specific context. This metabolic divergence underscores the urgent necessity for disease-specific precision nutrition strategies that prioritize metabolic resilience over generic restriction, offering a vital avenue for integrated oral–cardiovascular care that transitions clinical practice from a generalized framework to personalized medicine.

## Data Availability

The raw data supporting the conclusions of this article will be made available by the authors, without undue reservation.

## Glossary

CVD: Cardiovascular disease
NHANES: National Health and Nutrition Examination Survey
MPED: MyPyramid Equivalents Database
AUC-ROC: Area under the receiver operating characteristic curve
PD: Probing depth
AL: Attachment loss
HR: Hazard ratio
ROS: Reactive oxygen species
ML: Machine learning
SHAP: SHapley Additive exPlanations
LIME: Local Interpretable Model-agnostic Explanations
USDA: United States Department of Agriculture
CDC: Centers for Disease Control and Prevention
AAP: American Academy of Periodontology
BMI: Body mass index
HbA1c: Glycated hemoglobin
OGTT: Oral glucose tolerance test
SMOTE: Synthetic Minority Oversampling Technique
SVM: Support Vector Machine
XGBoost: eXtreme Gradient Boosting
AUC: Area under the curve
PR: Precision-Recall
OR: Odds ratio
CRP: C-reactive protein
TMAO: Trimethylamine N-oxide
SCFA: Short-chain fatty acids
eNOS: Endothelial nitric oxide synthase
HDL: High-density lipoprotein
