# Supplementary figures and images for: Uncovering non-linear dietary predictors of cardiovascular disease risk in older adults with periodontitis: a cross-sectional analysis
Source: Front Nutr. 2026 Mar 18;13:1791821. doi: 10.3389/fnut.2026.1791821 (PMC13038995; doi:10.3389/fnut.2026.1791821)

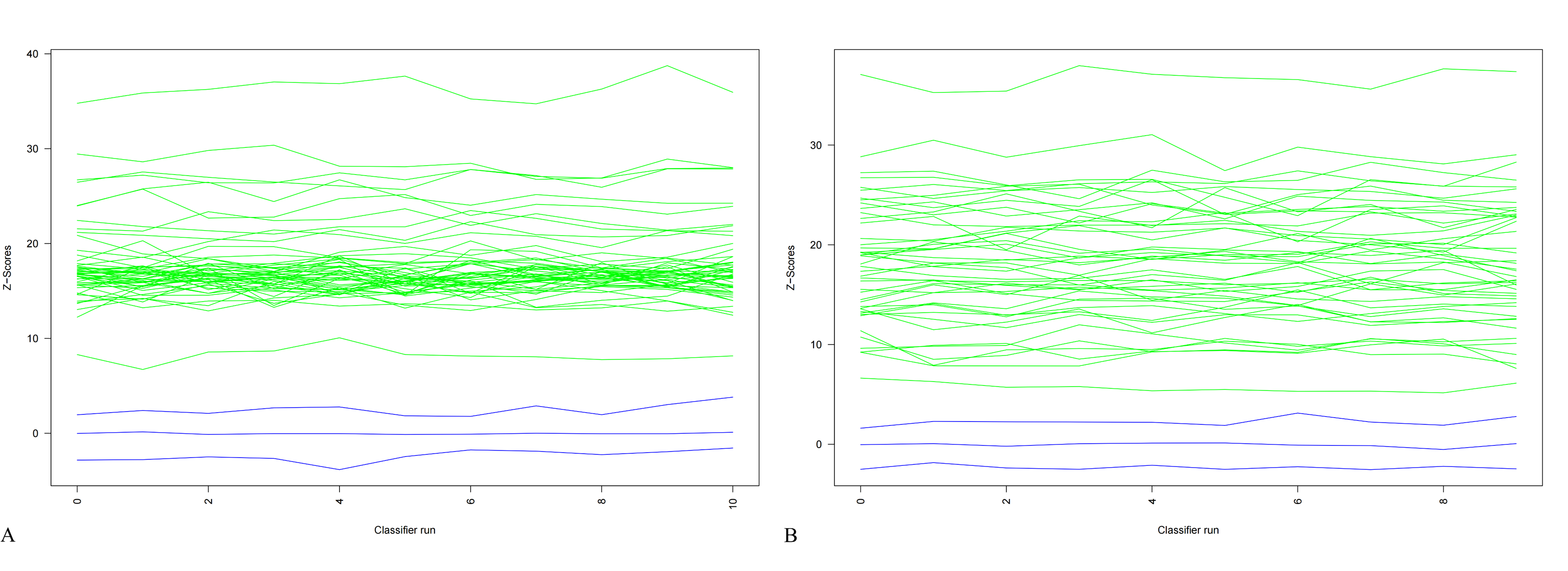

Supplement: Supplementary file 1 [file Image_1.tif]

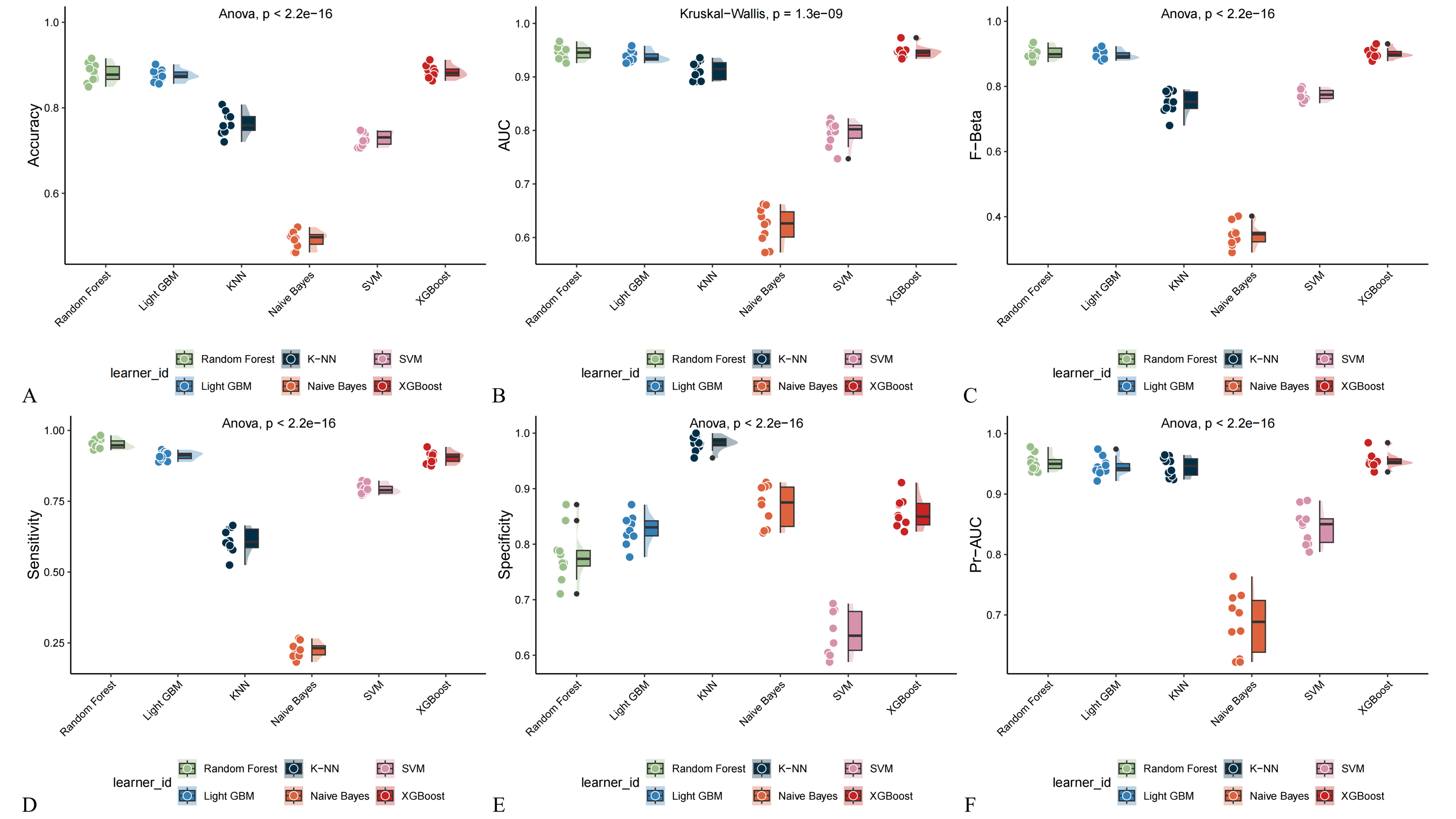

Supplement: Supplementary file 2 [file Image_2.tif]

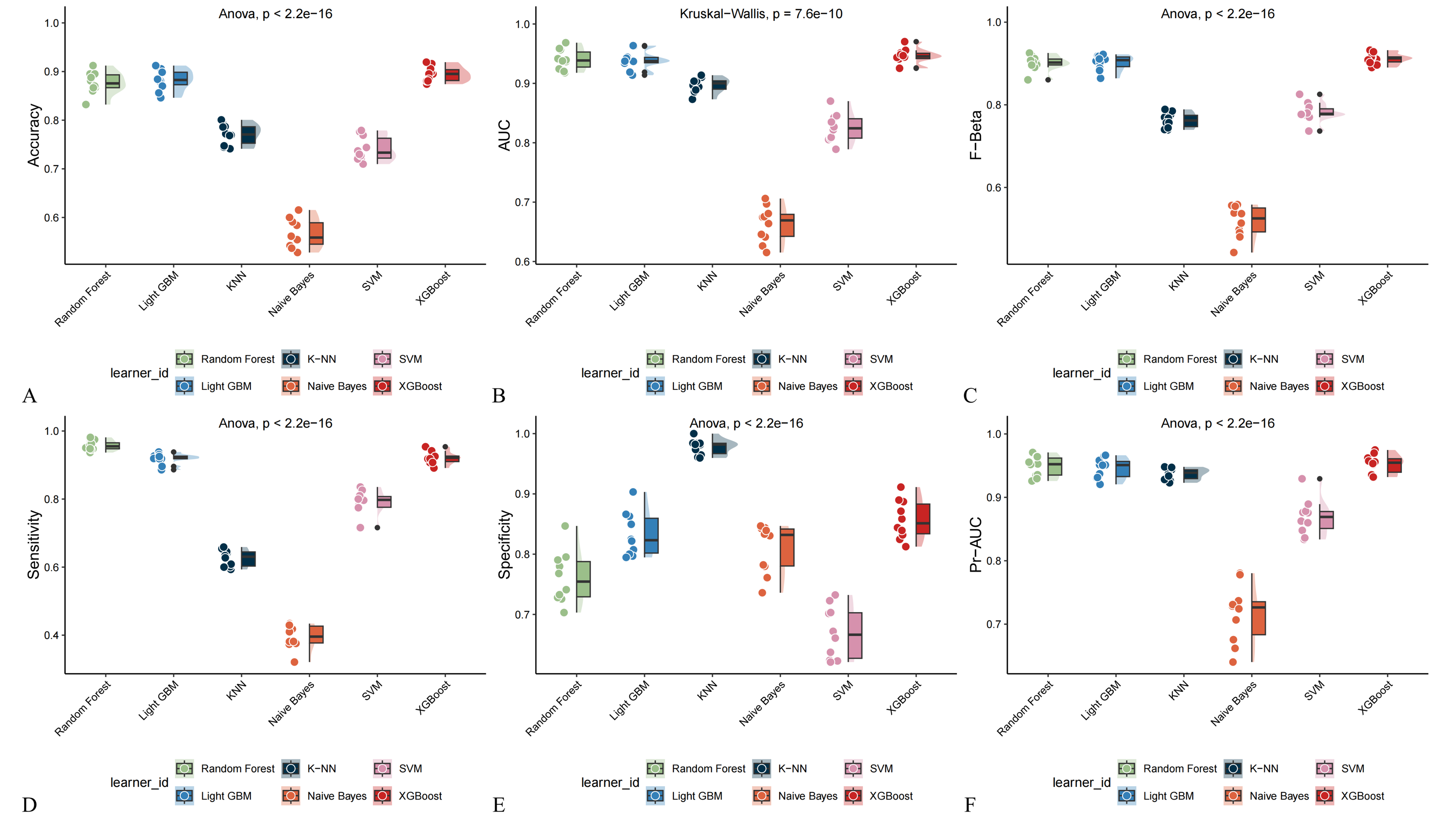

Supplement: Supplementary file 3 [file Image_3.tif]
