# Supplementary material for: Uncovering non-linear dietary predictors of cardiovascular disease risk in older adults with periodontitis: a cross-sectional analysis
Source: Front Nutr. 2026 Mar 18;13:1791821. doi: 10.3389/fnut.2026.1791821 (PMC13038995; doi:10.3389/fnut.2026.1791821)
Supplement: Supplementary file 4 [file Table_1.docx]

Supplementary Table 1. Detailed dietary micronutrient and food group intake profiles of older adults with periodontitis, stratified by CVD status.

| **Characteristic** | **N** | **Overall  (n =** **2,734)** | **Non-CVD  (n = 2,400)** | **CVD  (n = 334)** | ***P*** |
| --- | --- | --- | --- | --- | --- |
| Total Fat ^a^, Mean ± SD | 2,734 | 75.66 ± 44.26 | 76.05 ± 44.12 | 72.85 ± 45.23 | 0.153 |
| Saturated fatty acids ^a^, Mean ± SD | 2,734 | 24.18 ± 15.54 | 24.27 ± 15.59 | 23.55 ± 15.18 | 0.422 |
| Monounsaturated fatty acids ^a^, Mean ± SD | 2,734 | 27.43 ± 17.32 | 27.56 ± 17.30 | 26.47 ± 17.48 | 0.216 |
| Polyunsaturated fatty acids ^a^, Mean ± SD | 2,734 | 17.34 ± 11.54 | 17.46 ± 11.48 | 16.49 ± 11.96 | 0.063 |
| Cholesterol ^a^, Mean ± SD | 2,734 | 296.19 ± 245.80 | 297.37 ± 245.55 | 287.69 ± 247.80 | 0.403 |
| Vitamin E ^a^, Mean ± SD | 2,734 | 7.92 ± 6.11 | 7.97 ± 6.19 | 7.55 ± 5.51 | 0.186 |
| Alpha tocopherol ^a^, Mean ± SD | 2,734 | 0.49 ± 2.86 | 0.48 ± 2.88 | 0.60 ± 2.72 | 0.505 |
| Retinol ^a^, Mean ± SD | 2,734 | 424.42 ± 830.05 | 426.65 ± 869.49 | 408.42 ± 455.93 | 0.670 |
| Vitamin A ^a^, Mean ± SD | 2,734 | 643.88 ± 1,003.80 | 652.11 ± 1,051.67 | 584.78 ± 545.64 | 0.464 |
| Alpha carotene ^a^, Mean ± SD | 2,734 | 421.44 ± 1,677.69 | 420.40 ± 1,748.55 | 428.86 ± 1,036.31 | 0.763 |
| Beta carotene ^a^, Mean ± SD | 2,734 | 2,377.36 ± 6,054.99 | 2,448.75 ± 6,373.80 | 1,864.35 ± 2,815.41 | 0.210 |
| Beta cryptoxanthin ^a^, Mean ± SD | 2,734 | 101.95 ± 540.88 | 104.65 ± 575.11 | 82.56 ± 133.52 | 0.753 |
| Lycopene ^a^, Mean ± SD | 2,734 | 4,606.15 ± 8,627.20 | 4,576.14 ± 8,664.13 | 4,821.84 ± 8,366.58 | 0.386 |
| Lutein+zeaxanthin ^a^, Mean ± SD | 2,734 | 1,696.89 ± 4,005.65 | 1,767.16 ± 4,213.52 | 1,191.99 ± 1,869.02 | 0.006 |
| Vitamin B1 ^a^, Mean ± SD | 2,734 | 1.55 ± 0.83 | 1.56 ± 0.84 | 1.49 ± 0.77 | 0.189 |
| Vitamin B2 ^a^, Mean ± SD | 2,734 | 2.03 ± 1.18 | 2.03 ± 1.20 | 1.98 ± 1.06 | 0.537 |
| Niacin ^a^, Mean ± SD | 2,734 | 24.26 ± 13.46 | 24.36 ± 13.47 | 23.55 ± 13.42 | 0.144 |
| Vitamin B6 ^a^, Mean ± SD | 2,734 | 2.02 ± 1.36 | 2.03 ± 1.37 | 1.94 ± 1.33 | 0.067 |
| Total folate ^a^, Mean ± SD | 2,734 | 394.64 ± 240.23 | 398.45 ± 242.59 | 367.31 ± 220.98 | 0.011 |
| Folic acid ^a^, Mean ± SD | 2,734 | 169.98 ± 164.04 | 169.75 ± 164.23 | 171.65 ± 162.85 | 0.832 |
| Food folate ^a^, Mean ± SD | 2,734 | 224.71 ± 154.37 | 228.77 ± 158.55 | 195.55 ± 116.28 | <0.001 |
| Folate DFE ^a^, Mean ± SD | 2,734 | 513.70 ± 336.87 | 517.35 ± 338.50 | 487.48 ± 324.24 | 0.046 |
| Total choline ^a^, Mean ± SD | 2,734 | 345.15 ± 210.45 | 347.69 ± 211.34 | 326.87 ± 203.33 | 0.069 |
| Vitamin B12 ^a^, Mean ± SD | 2,734 | 5.11 ± 9.14 | 5.13 ± 9.55 | 5.00 ± 5.22 | 0.780 |
| Added vitamin B12 ^a^, Mean ± SD | 2,734 | 0.83 ± 2.14 | 0.81 ± 2.11 | 0.97 ± 2.36 | 0.139 |
| Vitamin C ^a^, Mean ± SD | 2,734 | 83.74 ± 96.34 | 84.13 ± 92.17 | 80.89 ± 122.28 | 0.052 |
| Vitamin K ^a^, Mean ± SD | 2,734 | 119.54 ± 346.07 | 123.52 ± 367.02 | 90.94 ± 107.38 | 0.017 |
| Calcium ^a^, Mean ± SD | 2,734 | 881.08 ± 550.15 | 886.19 ± 556.19 | 844.34 ± 503.84 | 0.356 |
| Phosphorus ^a^, Mean ± SD | 2,734 | 1,322.83 ± 642.04 | 1,335.08 ± 645.16 | 1,234.82 ± 612.90 | 0.007 |
| Iron ^a^, Mean ± SD | 2,734 | 14.58 ± 8.47 | 14.61 ± 8.49 | 14.38 ± 8.36 | 0.635 |
| Zinc ^a^, Mean ± SD | 2,734 | 10.99 ± 7.61 | 10.91 ± 6.65 | 11.57 ± 12.48 | 0.921 |
| Copper ^a^, Mean ± SD | 2,734 | 1.31 ± 1.60 | 1.32 ± 1.66 | 1.23 ± 1.11 | 0.040 |
| Sodium ^a^, Mean ± SD | 2,734 | 3,360.58 ± 1,703.68 | 3,375.24 ± 1,696.79 | 3,255.22 ± 1,751.44 | 0.113 |
| Potassium ^a^, Mean ± SD | 2,734 | 2,708.58 ± 1,283.01 | 2,730.50 ± 1,298.97 | 2,551.04 ± 1,151.40 | 0.032 |
| Selenium ^a^, Mean ± SD | 2,734 | 111.58 ± 65.77 | 112.12 ± 66.00 | 107.72 ± 64.07 | 0.067 |
| Caffeine ^a^, Mean ± SD | 2,734 | 163.41 ± 196.53 | 163.64 ± 194.40 | 161.72 ± 211.48 | 0.159 |
| Theobromine ^a^, Mean ± SD | 2,734 | 33.75 ± 79.66 | 33.83 ± 81.63 | 33.17 ± 63.87 | 0.375 |
| Alcohol ^a^, Mean ± SD | 2,734 | 10.23 ± 28.90 | 10.58 ± 29.28 | 7.68 ± 25.90 | 0.035 |
| Moisture ^a^, Mean ± SD | 2,734 | 2,773.30 ± 1,422.78 | 2,788.63 ± 1,428.13 | 2,663.12 ± 1,380.87 | 0.065 |
| Vitamin D ^a^, Mean ± SD | 2,734 | 4.88 ± 6.29 | 4.95 ± 6.50 | 4.41 ± 4.53 | 0.397 |
| Drinking status ^b^, n(%) | 2,734 |  |  |  | 0.703 |
| No |  | 387 (14.16%) | 342 (14.25%) | 45 (13.47%) |  |
| Yes |  | 2,347 (85.84%) | 2,058 (85.75%) | 289 (86.53%) |  |
| Borderline |  | 266 (9.73%) | 228 (9.50%) | 38 (11.38%) |  |
| Citrus, Melons, and Berries ^a^, Mean ± SD | 2,734 | 0.22 ± 0.65 | 0.22 ± 0.63 | 0.21 ± 0.72 | 0.527 |
| Other Intact Fruits ^a^, Mean ± SD | 2,734 | 0.58 ± 0.93 | 0.59 ± 0.94 | 0.54 ± 0.83 | 0.566 |
| Fruit Juices ^a^, Mean ± SD | 2,734 | 0.29 ± 0.69 | 0.30 ± 0.70 | 0.28 ± 0.62 | 0.916 |
| Total Fruits ^a^, Mean ± SD | 2,734 | 1.09 ± 1.40 | 1.10 ± 1.41 | 1.03 ± 1.32 | 0.508 |
| Dark Green Vegetables ^a^, Mean ± SD | 2,734 | 0.14 ± 0.41 | 0.15 ± 0.42 | 0.09 ± 0.30 | 0.003 |
| Tomatoes ^a^, Mean ± SD | 2,734 | 0.28 ± 0.44 | 0.28 ± 0.44 | 0.27 ± 0.41 | 0.489 |
| Other Red and Orange Vegetables ^a^, Mean ± SD | 2,734 | 0.10 ± 0.27 | 0.10 ± 0.28 | 0.09 ± 0.21 | 0.982 |
| Total Red and Orange Vegetables ^a^, Mean ± SD | 2,734 | 0.37 ± 0.52 | 0.37 ± 0.52 | 0.36 ± 0.45 | 0.696 |
| White Potatoes ^a^, Mean ± SD | 2,734 | 0.36 ± 0.62 | 0.35 ± 0.62 | 0.37 ± 0.60 | 0.639 |
| Other Starchy Vegetables ^a^, Mean ± SD | 2,734 | 0.10 ± 0.30 | 0.10 ± 0.29 | 0.11 ± 0.34 | 0.854 |
| Total Starchy Vegetables ^a^, Mean ± SD | 2,734 | 0.45 ± 0.70 | 0.45 ± 0.70 | 0.48 ± 0.72 | 0.613 |
| Other Vegetables ^a^, Mean ± SD | 2,734 | 0.56 ± 0.82 | 0.57 ± 0.84 | 0.49 ± 0.65 | 0.051 |
| Total Vegetables ^a^, Mean ± SD | 2,734 | 1.53 ± 1.38 | 1.54 ± 1.39 | 1.42 ± 1.24 | 0.097 |
| Legumes as Vegetables ^a^, Mean ± SD | 2,734 | 0.16 ± 0.42 | 0.16 ± 0.43 | 0.13 ± 0.36 | 0.832 |
| Refined Grains ^a^, Mean ± SD | 2,734 | 5.35 ± 3.81 | 5.38 ± 3.88 | 5.09 ± 3.25 | 0.582 |
| Total Grains ^a^, Mean ± SD | 2,734 | 6.25 ± 3.88 | 6.30 ± 3.94 | 5.88 ± 3.37 | 0.151 |
| Cured Meat ^a^, Mean ± SD | 2,734 | 0.96 ± 1.69 | 0.94 ± 1.67 | 1.07 ± 1.83 | 0.293 |
| Organ Meat ^a^, Mean ± SD | 2,734 | 0.03 ± 0.41 | 0.04 ± 0.43 | 0.03 ± 0.25 | 0.953 |
| Poultry ^a^, Mean ± SD | 2,734 | 1.49 ± 2.61 | 1.52 ± 2.63 | 1.27 ± 2.46 | 0.178 |
| Seafood High in n-3 ^a^, Mean ± SD | 2,734 | 0.18 ± 0.92 | 0.19 ± 0.94 | 0.12 ± 0.74 | 0.050 |
| Seafood Low in n-3 ^a^, Mean ± SD | 2,734 | 0.68 ± 2.39 | 0.67 ± 2.31 | 0.74 ± 2.93 | 0.244 |
| Total Meat Poultry Seafood ^a^, Mean ± SD | 2,734 | 4.94 ± 4.23 | 4.95 ± 4.20 | 4.88 ± 4.44 | 0.495 |
| Eggs ^a^, Mean ± SD | 2,734 | 0.60 ± 0.93 | 0.60 ± 0.94 | 0.58 ± 0.87 | 0.824 |
| Soy Products ^a^, Mean ± SD | 2,734 | 0.04 ± 0.28 | 0.04 ± 0.28 | 0.04 ± 0.25 | 0.597 |
| Nuts and Seeds ^a^, Mean ± SD | 2,734 | 0.69 ± 1.91 | 0.70 ± 1.97 | 0.56 ± 1.39 | 0.613 |
| Legumes as Protein Foods ^a^, Mean ± SD | 2,734 | 0.63 ± 1.69 | 0.64 ± 1.72 | 0.53 ± 1.45 | 0.817 |
| Total Protein Foods ^a^, Mean ± SD | 2,734 | 6.27 ± 4.83 | 6.30 ± 4.82 | 6.05 ± 4.92 | 0.183 |
| Milk ^a^, Mean ± SD | 2,734 | 0.76 ± 1.04 | 0.77 ± 1.06 | 0.69 ± 0.85 | 0.373 |
| Yogurt ^a^, Mean ± SD | 2,734 | 0.04 ± 0.16 | 0.04 ± 0.16 | 0.04 ± 0.15 | 0.340 |
| Cheese ^a^, Mean ± SD | 2,734 | 0.55 ± 0.88 | 0.55 ± 0.89 | 0.54 ± 0.85 | 0.858 |
| Total Dairy ^a^, Mean ± SD | 2,734 | 1.36 ± 1.39 | 1.37 ± 1.42 | 1.29 ± 1.21 | 0.618 |
| Oils ^a^, Mean ± SD | 2,734 | 22.65 ± 20.10 | 22.92 ± 20.28 | 20.73 ± 18.63 | 0.063 |
| Solid Fats ^a^, Mean ± SD | 2,734 | 34.23 ± 26.64 | 34.22 ± 26.70 | 34.31 ± 26.27 | 0.863 |
| a: Student t-test, b: Chi-square test, SD: standard deviation | | | | | |
