# Supplementary material for: Uncovering non-linear dietary predictors of cardiovascular disease risk in older adults with periodontitis: a cross-sectional analysis
Source: Front Nutr. 2026 Mar 18;13:1791821. doi: 10.3389/fnut.2026.1791821 (PMC13038995; doi:10.3389/fnut.2026.1791821)
Supplement: Supplementary file 5 [file Table_2.docx]

Supplementary Table 2. Benchmark comparison results for the NHANES training set.

| **Model** | **Accuracy** | **F Beta** | **Area under the ROC curve** | **Sensitivity** | **Specificity** | **Area under the PR curve** |
| --- | --- | --- | --- | --- | --- | --- |
| Random Forest | 0.868 (0.855-0.882) | 0.894 (0.881-0.907) | 0.946 (0.938-0.954) | 0.949 (0.938-0.959) | 0.752 (0.725-0.779) | 0.954 (0.943-0.966) |
| Light GBM | 0.867 (0.859-0.875) | 0.889 (0.881-0.897) | 0.938 (0.932-0.944) | 0.906 (0.898-0.914) | 0.812 (0.796-0.829) | 0.949 (0.941-0.957) |
| K-KNN | 0.765 (0.741-0.790) | 0.753 (0.725-0.780) | 0.905 (0.892-0.918) | 0.610 (0.574-0.646) | 0.988 (0.979-0.997) | 0.940 (0.931-0.950) |
| Naive Bayes | 0.492 (0.480-0.504) | 0.345 (0.323-0.366) | 0.624 (0.596-0.652) | 0.228 (0.210-0.246) | 0.868 (0.838-0.897) | 0.685 (0.648-0.723) |
| SVM | 0.714 (0.698-0.729) | 0.762 (0.747-0.778) | 0.787 (0.768-0.807) | 0.784 (0.767-0.800) | 0.613 (0.590-0.636) | 0.841 (0.822-0.860) |
| XGBoost | 0.886 (0.877-0.895) | 0.903 (0.894-0.912) | 0.949 (0.944-0.954) | 0.907 (0.891-0.923) | 0.855 (0.839-0.870) | 0.959 (0.952-0.966) |
| P | **<.001**^a^ | **<.001**^a^ | **<.001**^b^ | **<.001**^a^ | **<.001**^a^ | **<.001**^a^ |

a: ANOVA test; b: Kruskal-Wallis
