# Supplementary material for: Uncovering non-linear dietary predictors of cardiovascular disease risk in older adults with periodontitis: a cross-sectional analysis
Source: Front Nutr. 2026 Mar 18;13:1791821. doi: 10.3389/fnut.2026.1791821 (PMC13038995; doi:10.3389/fnut.2026.1791821)
Supplement: Supplementary file 6 [file Table_3.docx]

Supplementary Table 3. Benchmark comparison results for the MyPyramid Equivalents Database training set.

| **Model** | **Accuracy** | **F Beta** | **Area under the ROC curve** | **Sensitivity** | **Specificity** | **Area under the PR curve** |
| --- | --- | --- | --- | --- | --- | --- |
| Random Forest | 0.877 (0.862-0.892) | 0.901 (0.888-0.914) | 0.938 (0.929-0.948) | 0.958 (0.949-0.966) | 0.761 (0.731-0.792) | 0.945 (0.935-0.955) |
| Light GBM | 0.884 (0.867-0.902) | 0.904 (0.888-0.919) | 0.936 (0.926-0.947) | 0.925 (0.906-0.945) | 0.826 (0.797-0.854) | 0.943 (0.931-0.955) |
| K-KNN | 0.765 (0.748-0.782) | 0.755 (0.738-0.771) | 0.899 (0.889-0.909) | 0.615 (0.594-0.637) | 0.980 (0.972-0.988) | 0.940 (0.935-0.944) |
| Naive Bayes | 0.563 (0.542-0.585) | 0.514 (0.482-0.545) | 0.663 (0.630-0.695) | 0.394 (0.366-0.422) | 0.803 (0.764-0.842) | 0.711 (0.672-0.749) |
| SVM | 0.747 (0.726-0.767) | 0.789 (0.771-0.806) | 0.827 (0.804-0.850) | 0.805 (0.787-0.823) | 0.665 (0.624-0.705) | 0.868 (0.851-0.886) |
| XGBoost | 0.883 (0.871-0.896) | 0.902 (0.892-0.912) | 0.940 (0.930-0.951) | 0.916 (0.909-0.924) | 0.836 (0.812-0.860) | 0.948 (0.938-0.959) |
| P | **<.001**^a^ | **<.001**^a^ | **<.001**^b^ | **<.001**^a^ | **<.001**^a^ | **<.001**^a^ |

a: ANOVA test; b: Kruskal-Wallis
